# The Relationship between Plasma Vitamin D Concentration and Blood Pressure in Korean Middle-aged Males: A Cross-sectional Study

**Published:** 2018-11

**Authors:** Jooyoung KIM, Hyun-Jung PARK, Dong Jun SUNG

**Affiliations:** 1. Sports, Health, and Rehabilitation Major, College of Physical Education, Kookmin University, Seoul, Korea; 2. Dept. of Nursing, College of Social Services, Pyeongtaek University, Pyeongtaek, Gyeonggi, Korea; 3. Division of Sport and Health Science, College of Biomedical and Health Science, Konkuk University, Chungju, Chungbuk, Korea

## Dear Editor-in-Chief

In the case of Korean people, the intake of sodium added during the course of cooking food reaches 73%–80% of the daily recommended in-take (1) and taking into consideration the amount of sodium taken as food, it is more than in other countries, which indicates a susceptibility to hypertension. In addition, if the vitamin D intake is insufficient, the onset of hypertension can potentially increase.

The prevalence of vitamin D [25-hydroxyvitamin D 25(OH) D; a circulatory form of vitamin D] insufficiency in Korean males and females was 33.8% and 66.2%, respectively (2). Moreover, for males older than 49 yr old, the vitamin D deficiency level was reported to be more serious at 50.4% (3). In addition, vitamin D deficiency is a global public health problem not only in Korea. In general, a deficiency in vitamin D is known to cause hyperparathyroidism and subsequently bone loss through imbalance of the Ca^2+^ homeostasis (4).

Regarding the biological links between vitamin D and blood pressure, the conversion of 25(OH)D to 1,25-dihydroxy vitamin D [1,25(OH)_2_D] by 1α-hydroxylase is facilitated by Ca^2+^ influx into vascular smooth muscle cells, thereby inhibiting renin secretion in the juxtaglomerular cells of the kidney (5). Vitamin D may inhibit the renin-angiotensin system and subsequently prevent an elevation in blood pressure (6). Moreover, 25 (OH) D concentration in blood and vitamin D supplementation had a positive effect or correlation in preventing hypertension (7,8).

The concentration of vitamin D has a potent relationship with blood pressure. However, studies on the relationship between the blood levels of vitamin D and blood pressure in Koreans are poorly understood. Therefore, the aim of this study was to examine the relationship between vitamin D levels and blood pressure based on the sixth Korea National Health and Nutrition Examination Survey (KNHANES VI-2) (9).

In this study, 474 subjects were selected from 3885 males who met all of the following conditions. First, middle-aged men (40–60 yr), and second, those who measured blood pressure and 25(OH) D. The subjects had an average (mean ± standard deviation) age of 50.28 ± 8.95 yr, average height of 169.91±5.85 cm, average weight of 70.83±10.69 kg, average body mass index of 24.50±3.11 kg/m^2^, average 25(OH)D of 20.58 ± 8.95 ng/ml, and average systolic (SBP) and diastolic blood pressure (DBP) of 120.10±14.48 mmHg and 80.69±9.61 mmHg, respectively.

The correlations between vitamin D [25(OH) D] and blood pressure, including pulse pressure with the applied weight average were tested through the partial correlation coefficient test using SPSS version 19.0 for Windows (Chicago, IL, USA) with an adjusted weight, body mass index, physical activity level and Na^+^ intake. The level of significance was set at *P*<0.05.

**Fig. 1:**
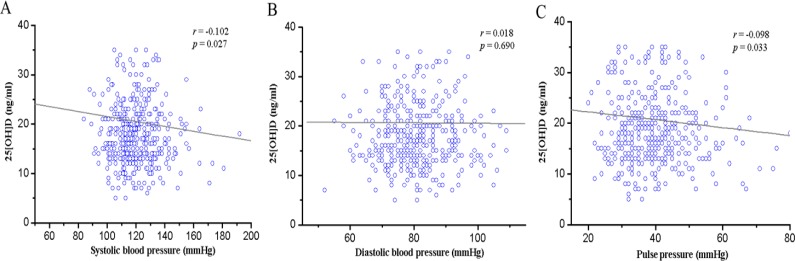
Correlation of 25(OH)D with systolic blood pressure, diastolic blood pressure, and pulse pressure. A. 25(OH)D with systolic blood pressure (*r* = −0.102, *P*=0.027). B, 25(OH)D with diastolic blood pressure (*r* = 0.018, *P*=0.690). C, 25(OH)D with pulse pressure (*r* = −0.098, *P*=0.033)

25(OH) D was negatively correlated with SBP (*r*=−0.102, *P*=0.027). In contrast to SBP, DBP had no a relationship with 25(OH) D (*r*=0.018, *P*=0.690). In pulse pressure and 25(OH) D, significant negative correlation was observed (*r*=−0.098, *P*=0.033) (Fig. 1). Thus, level of 25(OH) D might be factors that affect blood pressure in middle-aged males. Therefore, an increase in the intake of vitamin D may be an effective way to prevent the elevation of blood pressure. In addition, our results showed a significant correlation between vitamin D and systolic blood pressure, but it is hard to argue that vitamin D is a major factor in preventing an elevation in the blood pressure. Various factors, including regular exercise, eating habits, and other risk factors should be considered to prevent hypertension.
